# Early findings in a randomised controlled trial on crosslinking protocols using isoosmolar and hypoosmolar riboflavin for the treatment of progressive keratoconus

**DOI:** 10.1111/aos.16736

**Published:** 2024-07-05

**Authors:** Ingemar Gustafsson, Thorbjörg Olafsdottir, Olof Neumann, Per Johansson, Dimitrios Bizios, Anders Ivarsen, Jesper Ø. Hjortdal

**Affiliations:** ^1^ Department of Ophthalmology Skåne University Hospital Malmö Sweden; ^2^ Department of Clinical Sciences Lund University Lund Sweden; ^3^ Department of Clinical Sciences Lund University Malmö Sweden; ^4^ Department of Ophthalmology Aarhus University Hospital Aarhus Denmark

**Keywords:** corneal crosslinking, keratoconus, progressive keratoconus, riboflavin

## Abstract

**Purpose:**

To present baseline characteristics and to present the perioperative corneal thickness during corneal crosslinking (CXL) treatment for progressive keratoconus and to describe how the addition of sterile water (SW) efficaciously can maintain the corneal thickness. The treatment efficacy will be evaluated when the 1‐year follow‐up is complete.

**Methods:**

A randomised clinical study using epithelium‐off CXL with continuous UVA irradiation (9 mW/cm^2^) and two kinds of riboflavin solutions: (i) isoosmolar dextran‐based riboflavin (*n* = 27) and (ii) hypoosmolar dextran‐free riboflavin (*n* = 27). Inclusion criteria: progressive keratoconus with an increase in maximum keratometry value (Kmax) of 1.0 dioptre (12 months) or 0.5 dioptres (6 months). Corneae thinner than 400 μm were also included. Outcome parameters: Perioperative corneal thickness and the effect of adding SW.

**Results:**

Seventy‐four per cent of the patients in the isoosmolar group and 15% in the hypoosmolar group required the addition of SW, which effectively maintained a corneal thickness of 400 μm in all cases during CXL. The addition of SW was primarily needed during the irradiation procedure and not the preoperative soaking period.

**Conclusions:**

Especially during the CXL irradiation phase, isoosmolar riboflavin causes a significant dehydrating effect leading to corneal thinning during CXL. The customised addition of SW is efficacious in maintaining the corneal thickness during CXL and could increase the safety of the procedure.

## 1 INTRODUCTION

Corneal crosslinking (CXL) is performed to arrest the progression of keratoconus disease and was introduced by Wollensak et al. approximately 20 years ago (Wollensak et al., 2003). Over 1000 such treatments are performed each year in the Nordic countries (Gustafsson, Vicente, et al., 2023), and the long‐term efficacy of CXL in preventing further disease progression has been demonstrated (Knutsson et al., 2023; Raiskup‐Wolf et al., 2008) which has contributed to a reduction in the number of corneal transplantations (Sandvik et al., 2015). However, according to Cochrane authors, more randomised clinical trials (RCTs) are needed to optimise and evaluate both existing and novel CXL techniques (Ng et al., 2021; Sykakis et al., 2015).

This RCT (NCT04427956) was designed to evaluate the efficacy in halting keratoconus disease progression using continuous 9 mW/cm^2^ UVA (365 nm) irradiance in three different treatment arms based on the type of riboflavin and the modality of riboflavin delivery: (i) an epithelium‐off technique using dextran‐based isoosmolar riboflavin, (ii) an epithelium‐off technique using dextran‐free hypoosmolar riboflavin and (iii) an epithelium‐on technique using iontophoresis‐assisted delivery of riboflavin. Treatment with iontophoresis‐assisted delivery of riboflavin was interrupted due to its low efficacy in halting disease progression, and the outcomes of this arm of the study have been published elsewhere (Gustafsson, Ivarsen, & Hjortdal, 2023).

Most centres in the Nordic countries use continuous UVA irradiation of 9 mW/cm^2^ (Gustafsson, Vicente, et al., 2023) which is also in accordance with a recently published survey on clinical practice regarding CXL in the United Kingdom (Hayes et al., 2023). Dextran‐based riboflavin is the original form of riboflavin used in the Dresden Protocol (Wollensak et al., 2003) and is the most frequently used type of riboflavin in clinical trials (Kuo et al., 2021; Sykakis et al., 2015). However, the oncotic effect of this form of riboflavin (Mazzotta & Caragiuli, 2014) in association with evaporation of the stromal water (Iwata et al., 1969) from the de‐epithelialised cornea can cause a reduction in corneal thickness (Kymionis et al., 2009; Rosenblat & Hersh, 2016) to below the suggested safe limit of 400 μm, thus exposing the endothelial cells to excessive radiation (Seiler et al., 2019; Spoerl et al., 2007). We therefore used hypoosmolar riboflavin in the second treatment arm, as it has been suggested that this causes less reduction in corneal thickness, thus rendering more patients eligible for safe CXL (Hafezi et al., 2009). We also investigated the need to add sterile water (SW) during the CXL procedure and the effects this had on the efficacy and safety of CXL.

Hence, the purpose of this study was to assess the safety and treatment efficacy of hypoosmolar and isoosmolar riboflavin when using continuous UVA irradiation of 9 mW/cm^2^ for 10 min. Although hypoosmolar riboflavin is commonly used in clinical practice in Nordic countries (Gustafsson, Vicente, et al., 2023), no previous randomised clinical trials have been reported using hypoosmolar riboflavin in combination with an irradiation of 9 mW/cm^2^. Enrolment of the patients in this study was recently completed. In this paper, we describe the baseline characteristics of the enrolled patients and the intraoperative effect of the two riboflavins on the corneal thickness and the counteraction of excessive thinning by the use of SW. The final results regarding treatment efficacy and safety will be presented at the end of 2024.

## 2 SUBJECTS AND METHODS

The study is being conducted at the Department of Ophthalmology at Skåne University Hospital, Lund, Sweden, according to the tenets of the Declaration of Helsinki. All participants were given written information on the study, and written consent was obtained. The Regional Ethics Committee in Lund, Sweden, approved the study (No. 2015/373).

### 2.1 Study design

Randomised clinical trial (NCT04427956) https://clinicaltrials.gov.

### 2.2 Patient enrolment and randomisation

Patient enrolment has been described in a previous publication (Gustafsson, Ivarsen, & Hjortdal, 2023). Patients fulfilling the inclusion criteria were enrolled consecutively from January 2017 until 2023. The inclusion criteria were progressive keratoconus in patients aged ≥18 years. Progression was defined as an increase of 1.0 D in Kmax during the past 12 months or 0.5 D during the past 6 months and a history of deteriorating visual acuity (perceived or objective). Patients with a history of ocular pathology and ocular surgery were excluded, as were pregnant and breastfeeding women. Contact lens wear was discontinued at least 2 weeks before tomographic measurements were performed. A preoperative corneal thickness of <400 μm was not an exclusion criteria in order not to bias the recruitment and knowing the thickness could be modified to >400 μm.

Sealed envelopes were prepared by Clinical Studies Sweden, a national facility supported by the Swedish Research Council. An envelope was opened after each patient agreed to participate in the study. Only one eye per participating patient was included to avoid possible paired‐organ bias.

### 2.3 Pretreatment assessment

The pre‐CXL assessment consisted of measurements of uncorrected visual acuity (UCVA) and best spectacle‐corrected visual acuity (BSCVA) by opticians with long experience of refraction in keratoconus patients. Measurements were performed with a corneal tomographer (Pentacam HR, Oculus GmbH, Germany) followed by full confocal scanning of the cornea, in addition to an automated measurement of endothelial cell density (ECD) (Cofoscan 4, Nidek Technologies Srl, Italy). Furthermore, a slit lamp examination was performed.

### 2.4 CXL procedure

A drop of povidone iodine (Minims 5% eye drops, Bausch + Lomb, Ireland) was instilled in the eye followed by local anaesthesia (Tetracaine 1% eye drops, Bausch + Lomb, Ireland). An eyelid speculum was positioned, and the corneal surface was flushed with balanced salt solution (BSS). An 8 mm circular Merocel sponge (Merocel Corneal light shield, Beaver‐Visitec Int., Waltham, MA, USA) soaked in 35% ethanol was applied for 20 s, and epithelial removal was performed with a surgical cellulose sponge. Isoosmolar riboflavin (MedioCROSS D, Avedro, Inc., Waltham, Massachusetts, MA, USA) or hypoosmolar riboflavin (MedioCROSS H, Avedro, Inc., Waltham, Massachusetts, MA, USA) was instilled every 3 min during 20 min prior to irradiation. No eye lid speculum was used during the instillation phase of CXL. Corneal thickness measurements were obtained by pachymetry (Tomey SP‐100, Tomey Corporation, Nagoya, Japan) immediately after epithelial removal and then every 5 min during treatment. If the corneal thickness approached, or was below, 400 μm, SW (Sterile water, Braun AG, Melsungen, Germany) was added and additional measurements were performed after 1 min. After the instillation phase, the cornea was irradiated with UVA (UV‐X 2000, IROC, Switzerland) using an irradiation rate of 9 mW/cm^2^ for 10 min (total fluence 5.4 J/cm^2^). When the measurements were obtained and when SW was added, the irradiation was temporarily paused; however, the same total energy was delivered. Riboflavin instillation continued every third minute during UVA irradiation. The addition of SW was customised for each patient and therefore not standardised. At the end of the treatment, a drop of levofloxacin, 5 mg/mL (Oftaquix, Santen Oy, Finland), was applied. After CXL, levofloxacin (5 mg/mL) was prescribed four times a day for 7 days as a prophylaxis to prevent infection. In addition, a lubricating carbomer (Oftagel, 2.5 mg/g, Santen Oy, Finland) was prescribed four times a day for 1 month for comfort. A soft bandage lens (Air Optix Night and Day, Alcon, Fort Worth, Texas, USA) was applied for the first week in the first 15 patients to reduce pain, but as one patient developed bacterial keratitis, no contact lens was applied in the remaining 39 patients.

### 2.5 Post‐treatment assessment

The patients returned to the clinic for follow‐up visits after 7 days and after 1, 6, 12 and 24 months. This paper considers the perioperative results and the results after the first week. The purpose of the first visit after 7 days was to confirm healing of the corneal epithelium and to exclude signs of infection. No further measurements were performed at this visit.

### 2.6 Statistical analysis

This randomised study was designed as a non‐inferiority study with dextran‐based isoosmolar riboflavin as the reference protocol. A null difference between the reference protocol and the other two protocols was set at 1.0 D in Kmax, and a standard deviation of 1.2 D was assumed (alpha = 0.025; power = 0.81), resulting in a sample size of twenty‐four patients in each group, with an allocation rate of 1:1:1. Bonferroni correction was applied. A discontinuation rate of 10% was anticipated, giving a sample size of twenty‐seven patients in each group. SAS Enterprise Guide 6.1 for Windows (SAS Institute Inc., Cary, North Carolina, USA) was used to calculate the sample size.

Descriptive statistics are given as subject mean, standard deviation, median, minimum and maximum values and interquartile range.

## 3 RESULTS

The baseline characteristics of the patients are presented in Table 1. No significant differences were found between the groups. The mean progression in Kmax was numerically higher in the hypoosmolar group (3.1 D, range 0.5–19.1 D) than in the isoosmolar group (1.9 D, range 0–7.5 D), which is explained by one patient having an extreme progression of 19.1 D. The “0” in the range in the isoosmolar cohort reveals that one patient was erroneously recruited. Values of the endothelial cell density are missing for four patients due to low quality of the images caused by advanced keratoconus, and in one case, the patient declined to be examined. Preoperative progression in keratometric and tomographic parameters is described in Table 2.

**TABLE 1:** Baseline characteristics and the addition of sterile water during CXL.

	All patients	Isoosmolar	Hypoosmolar
	*n* = 54	*n* = 27	*n* = 27
Age (years)			
Mean (SD)	26.1 (5.1)	25.3 (4.9)	27.0 (5.3)
Median [Min, Max]	25.5 [18.0, 38.0]	24.0 [18.0, 35.0]	27.0 [18.0, 38.0]
Gender			
Male, *n* (%)	48 (88.9)	24 (88.9)	24 (88.9)
Female, *n* (%)	6 (11.1)	3 (11.1)	3 (11.1)
Eye			
Right, *n* (%)	26 (48.1)	12 (44.4)	14 (51.9)
Left, *n* (%)	28 (51.9)	15 (55.6)	13 (48.1)
ECD (cells/mm^2^)			
Mean (SD)	2582 (243)	2611 (234)	2554 (254)
Median [Min, Max]	2569 [2009, 3040]	2627 [2009, 3029]	2514 [2107, 3040]
Missing	5 (9.3)	3 (11.1)	2 (7.4)
Progression in Kmax (D)			
Mean (SD)	2.5 (3.0)	1.9 (1.6)	3.1 (3.8)
Median [Min, Max]	1.8 [0.0, 19.1]	1.5 [0.0, 7.5]	2.1 [0.50, 19.1]
Addition of SW			
No, *n* (%)	28 (51.9)	7 (25.9)	21 (77.8)
Yes, *n* (%)	24 (44.4)	20 (74.1)	4 (14.8)
Missing	2 (3.7)	0 (0.0)	2 (7.4)
Corneal thickness after epithelial debridement (μm)			
Mean (SD)	454 (44.3)	457 (40.6)	451 (48.3)
Median [Min, Max]	456 [321, 558]	454 [396, 558]	458 [321, 523]
SE			
Mean (SD)	−1.9 (3.1)	−1.7 (3.3)	−2.2 (2.8)
Median [Min, Max]	−1.6 [−11.1, 3.5]	−1.0 [−11.1, 3.5]	−1.8 [−9.0, 2.5]
BSCVA			
Mean (SD)	0.65 (0.27)	0.65 (0.28)	0.64 (0.26)
K2 (D)			
Mean (SD)	48.7 (4.76)	48.3 (3.91)	49.0 (5.53)
Median [Min, Max]	48.2 [41.9, 62.3]	48.4 [42.9, 57.2]	48.2 [41.9, 62.3]
Kmax (D)			
Mean (SD)	56.1 (7.69)	55.8 (6.64)	56.4 (8.73)
Median [Min, Max]	56.1 [43.6, 77.8]	56.8 [43.6, 66.4]	53.1 [46.0, 77.8]
A (mm)			
Mean (SD)	6.76 (0.679)	6.82 (0.628)	6.69 (0.733)
Median [Min, Max]	6.90 [4.95, 8.04]	6.78 [5.70, 8.04]	6.92 [4.95, 7.66]
B (mm)			
Mean (SD)	5.10 (0.596)	5.13 (0.559)	5.06 (0.640)
Median [Min, Max]	5.12 [3.75, 6.49]	5.12 [4.32, 6.49]	5.12 [3.75, 6.07]
C (μm)			
Mean (SD)	473 (48.1)	473 (48.9)	474 (48.3)
Median [Min, Max]	476 [369, 576]	470 [384, 576]	494 [369, 540]

*Note*: All measurements were made immediately prior to corneal crosslinking, and the use of sterile water was documented during the treatment procedure. A = anterior curvature of the 3 mm zone over the thinnest point (mm), B = posterior curvature of the 3 mm zone under the thinnest point (mm) and C = thickness of the thinnest point on the cornea (μm). The A, B and C parameters are from the Belin ABCD Progression Display Missing data: in two subjects in the hypoosmolar group, the use of SW was not correctly documented and these subjects were therefore excluded. In five patients, the ECD is missing. Values of the endothelial cell density are missing for four patients due to low quality of the images caused by advanced keratoconus, and in one case, the patient declined examination.

Abbreviations: BSCVA, best spectacle‐corrected visual acuity; D, dioptres; ECD, endothelial cell density; K2, steepest central keratometry reading; Kmax, maximum keratometric value; SD, standard deviation; SE, spherical equivalent; SW, sterile water; μm, micrometre.

**TABLE 2:** Progression in keratometric and tomographic values for the patients.

	Overall	Isoosmolar	Hypoosmolar
	*n* = 54	*n* = 27	*n* = 27
ΔK2 (D)			
Mean (SD)	1.17 (1.59)	1.14 (1.74)	1.19 (1.46)
Median [Min, Max]	0.600 [0, 8.50]	0.600 [0, 8.50]	0.600 [0, 5.90]
Missing, *n* (%)	3 (5.6)	2 (7.4)	1 (3.7)
ΔKmax (D)			
Mean (SD)	2.48 (2.98)	1.88 (1.64)	3.08 (3.82)
Median [Min, Max]	1.75 [0, 19.1]	1.50 [0, 7.50]	2.10 [0.500, 19.1]
ΔA (mm)			
Mean (SD)	−0.218 (0.291)	−0.171 (0.141)	−0.257 (0.373)
Median [Min, Max]	−0.140 [−1.90, −0.0100]	−0.125 [−0.670, 0.0500]	−0.155 [−1.90, −0.0100]
Missing, *n* (%)	10 (18.5)	7 (25.9)	3 (11.1)
ΔB (mm)			
Mean (SD)	−0.208 (0.329)	−0.149 (0.112)	−0.258 (0.432)
Median [Min, Max]	−0.125 [−2.10, 0.0900]	−0.145 [−0.440, 0.0500]	−0.115 [−2.10, 0.0900]
Missing, *n* (%)	10 (18.5)	7 (25.9)	3 (11.1)
ΔC (μm)			
Mean (SD)	−8.46 (14.7)	−9.55 (11.5)	−7.66 (16.8)
Median [Min, Max]	−6.75 [−66.0, 20.0]	−8.63 [−47.0, 6.00]	−6.00 [−66.0, 20.0]
Missing, *n* (%)	7 (13.0)	7 (25.9)	0 (0.0)

*Note*: Differences between a previous measurement (within a year) and the measurement at the moment of enrolment. A positive value for K2 and Kmax indicates progression, while a negative value indicates progression for the A, B and C parameters. ΔK2 = difference in the central steepest keratometry reading, ΔKmax = difference in the maximum keratometry reading and ΔA, ΔB, ΔC = difference in the magnitude of the A, B and C parameters in the Belin ABCD Progression Display. Missing data: The ABC data could not be analysed in ten patients due to referral from other centres, and the ABC data were thus not available.

Abbreviations: D, dioptres; SD, standard deviation.

### 3.1 Corneal thinning during the CXL procedure

**FIGURE 1:**
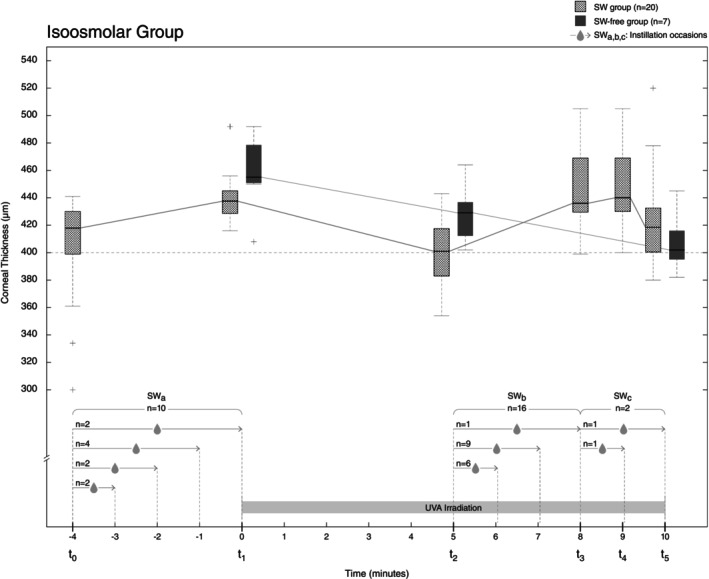
Boxplots of corneal thickness in micrometres (μm) for the isoosmolar group (*n* = 27) before and during the UVA irradiation phase of the CXL procedure. There were 3 occasions of sterile water instillation (SW_a,b,c_), lasting between one and 5 min, before the start and during the UVA irradiation phase. Twenty patients required sterile water (SW group) and 7 patients did not (SW‐free group). Riboflavin solution was instilled every 3 min for 20 min prior to CXL and also every 3 min during UVA irradiation. t0 denotes the end of riboflavin instillation, 4 min prior to the start of UVA irradiation. t1 denotes the start of irradiation and measurements made at t2 (5 min) and t5 (10 min, end of irradiation). Extra measurements were performed at t3 (8 min) and t4 (9 min) in those patients requiring SW instillation. The length of the arrows corresponds to the length of instillation time.

**FIGURE 2:**
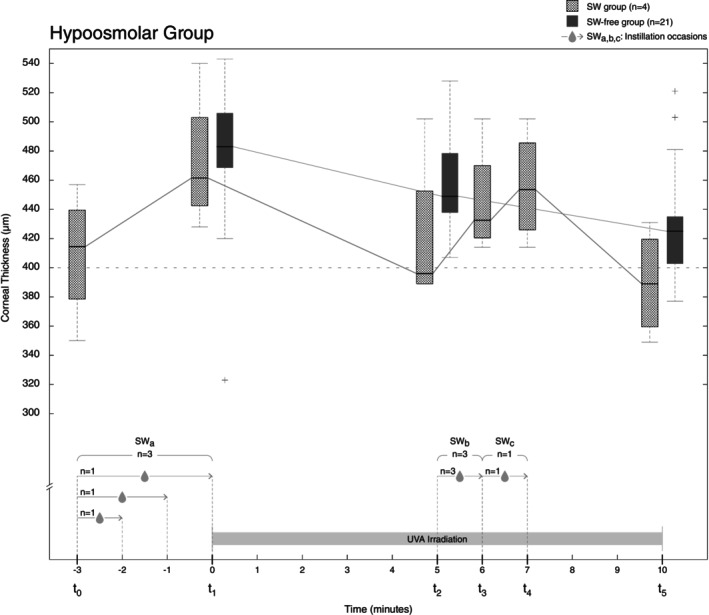
Boxplots of corneal thickness in micrometres (μm) for the hypoosmolar group (*n* = 25) before and during the UVA irradiation phase of the CXL procedure. There were 3 occasions of sterile water instillation (SW_a,b,c_), lasting between one and 3 min, before the start and during the UVA irradiation phase. Four patients required sterile water (SW group) and twenty‐one patients did not (SW‐free group). Riboflavin solution was instilled every 3 min for 20 min prior to CXL and also every 3 min during UVA irradiation. t0 denotes the end of riboflavin instillation, 3 min prior to the start of UVA irradiation. t1 denotes the start of irradiation and measurements made at t2 (5 min) and t5 (10 min, end of irradiation). Extra measurements were performed at t3 (6 min) and t4 (7 min) in those patients requiring SW instillation. The length of the arrows corresponds to the length of instillation time.

The effect of the two kinds of riboflavin on the corneal thickness during CXL is illustrated in Figure 1 (isoosmolar group) and 2 (hypoosmolar group). The measurements of corneal thickness in two patients in the hypoosmolar group did not follow the protocol and were thus excluded, resulting in 25 remaining. The values of the keratometric and tomographic parameters for the isoosmolar and hypoosmolar cohorts are given in Tables 3 and 4. In the isoosmolar cohort, 74% of the patients required the addition of SW, while the corresponding fraction in the hypoosmolar cohort was only 15%. Those requiring the addition of SW had more advanced keratoconus than those not requiring SW. The 7 patients in the isoosmolar cohort who did not require the addition of SW had a corneal thickness (before de‐epithelialisation) ranging from 470 to 576 μm with a mean value of 519 μm, compared to those requiring the addition of SW in the hypoosmolar group who had a range of 399–421 μm with a mean value of 456 μm. Figure 1 shows that most patients required the addition of SW during the irradiation phase compared to the instillation phase. Patients in the hypoosmolar cohort requiring the addition of SW already had corneae with a thickness below or close to the 400 μm safety limit prior to CXL with a range of 399–421 μm and a mean value of 408 μm. In fact, Figure 2 shows that 3 patients required the addition of SW both prior to CXL and during CXL, while a fourth patient required the addition of SW during CXL only. This is explained by the fact that the corneae were thin from the beginning. Those who did not require the addition of SW had a range of 369–540 μm with a mean value of 485 μm.

**TABLE 3:** Preoperative values for topographic and tomographic parameters for patients in the isoosmolar riboflavin group with and without the addition of sterile water during CXL.

	Isoosmolar riboflavin	Isoosmolar riboflavin + SW
	*n* = 7	*n* = 20
K2 (D)		
Mean (SD)	45.2 (2.6)	49.4 (3.7)
Median [q1, q3]	43.8 [43.4, 48.7]	49.1 [46.6, 51.1]
[Min, Max]	[43.4, 49.2]	[42.9, 57.2]
Kmax (D)		
Mean (SD)	50.5 (5.9)	57.7 (5.9)
Median [q1, q3]	47.6 [46.0, 56.8]	58.2 [52.5, 62.0]
[Min, Max]	[43.6, 58.2]	[46.0, 66.4]
A (mm)		
Mean (SD)	7.3 (0.67)	6.6 (0.52)
Median [q1, q3]	7.5 [6.5, 7.9]	6.5 [6.3, 7.1]
[Min, Max]	[6.2, 8.0]	[5.7, 7.7]
B (mm)		
Mean (SD)	5.5 (0.68)	5.0 (0.44)
Median [q1, q3]	5.5 [5.0, 6.2]	4.8 [4.7, 5.4]
[Min, Max]	[4.5, 6.5]	[4.3, 5.9]
C (mm)		
Mean (SD)	519 (47)	456 (39)
Median [q1, q3]	503 [477, 568]	465 [421, 496]
[Min, Max]	[470, 576]	[384, 514]

*Note*: All measurements were made immediately prior to corneal crosslinking. A = anterior curvature of the 3 mm zone over the thinnest point (mm), B = posterior curvature of the 3 mm zone under the thinnest point (mm), C = thickness of the thinnest point on the cornea (μm).

Abbreviations: D, dioptres; K2, steepest central keratometric value; Kmax, maximum keratometry value; SW, sterile water; SD, standard deviation; q, quartile.

**TABLE 4:** Preoperative values for topographic and tomographic parameters for patients in the hypoosmolar riboflavin group with and without the addition of sterile water during CXL.

	Hypoosmolar riboflavin	Hypoosmolar riboflavin + SW
	*n* = 23	*n* = 4
K2 (D)		
Mean (SD)	49.0 (5.5)	49.1 (6.3)
Median [q1, q3]	48.2 [45.9, 50.6]	48.6 [43.5, 55.2]
[Min, Max]	[42.3, 62.3]	[41.9, 57.2]
Kmax (D)		
Mean (SD)	49.0 (5.5)	60.6 (5.2)
Median [q1, q3]	48.2 [45.9, 50.6]	58.4 [57.4, 66.0]
[Min, Max]	[42.3, 62.3]	[57.4, 68.2]
A (mm)		
Mean (SD)	6.8 (0.75)	6.3 (0.54)
Median [q1, q3]	7.0 [6.2, 7.3]	6.3 [5.8, 6.8]
[Min, Max]	[5.0, 7.7]	[5.7, 7.0]
B (mm)		
Mean (SD)	5.1 (0.66)	4.8 (0.53)
Median [q1, q3]	5.2 [4.7, 5.6]	4.7 [4.4, 5.3]
[Min, Max]	[3.8, 6.1]	[4.3, 5.6]
C (μm)		
Mean (SD)	485 (43)	408 (11)
Median [q1, q3]	496 [459, 516]	406 [399, 419]
[Min, Max]	[369, 540]	[399, 421]

*Note*: All measurements were made immediately prior to corneal crosslinking. A = anterior curvature of the 3 mm zone over the thinnest point (mm), B = posterior curvature of the 3 mm zone under the thinnest point (mm), C = thickness of the thinnest point on the cornea (μm).

Abbreviations: D, dioptres; K2, steepest central keratometric value; Kmax, maximum keratometry value; SW, sterile water; SD, standard deviation; q, quartile.

### 3.2 Treatment‐associated side effects within the first week

All patients attended the follow‐up visit at 7 days, at which time all had an intact epithelium, although some showed signs of dry eyes. One patient had clinical signs of superficial bacterial keratitis measuring approximately 1 mm. Levofloxacin was replaced by chloramphenicol 10 mg/g (Chloramphenicol, Santen Oy, Finland) and tobramycin 3 mg/mL (Tobrex, Alcon, Sweden). The keratitis healed without any scar formation.

## 4 DISCUSSION

The purpose of this study was to assess the safety and treatment efficacy of hypoosmolar and isoosmolar riboflavin when using a continuous UVA irradiation rate of 9 mW/cm^2^ for 10 min.

Enrolment in the study is now completed, and early data from the treatment occasion are now available for all participants. The results regarding long‐term efficacy and safety will be presented when the 1‐year follow‐up is complete.

Riboflavin acts as a photoreactor when irradiated by UVA and induces free radicals followed by the formation of covalent crosslinks in the stroma (Sharif et al., 2018; Spoerl et al., 2007). It also provides protection against excessive photochemical damage, which could otherwise harm the intraocular structures (Spoerl et al., 2007). In fact, a safety limit of 400 μm to protect the endothelium has been suggested including a safety margin of 50 μm (Spoerl et al., 2007) based on the cytotoxic threshold of 0.36 mW/cm^2^ for the endothelial cells (Wollensak, 2010). The cytotoxic CXL‐related threshold for keratocytes was determined to be at 0.5 mW/cm^2^ (Wollensak, 2010). The clinical demarcation line (DL) correlates with the stromal depth of the CXL‐related keratocyte damage allowing a certain risk evaluation for the endothelium in relation to the distance of the DL from the endothelium. The DL also typically varies according to different CXL protocols reflecting differences in CXL efficacy (Mazzotta et al., 2019). The 400 μm safety limit is commonly used, although it has been suggested that it is unnecessarily conservative (Seiler et al., 2019). However, apart from ensuring the safety of the endothelial cells, a safety limit should also serve to avoid overcrosslinking (Seiler, 2021). Another serious cause for an endothelial damage is a defocus of the irradiation device. As little is known on this topic, we decided to use the 400 μm limit in this study.

The results demonstrate that isoosmolar riboflavin reduces the corneal thickness significantly during CXL, as has been described previously (Schmidinger et al., 2013). Although the isoosmolar riboflavin has an osmolarity of 402.7 mOsmL/L (Wollensak et al., 2003), similar to the osmolarity of the cornea, of approximately 420 mOsmL/L (Schrage et al., 2000), the hydrophilic dextran molecule has an oncotic effect, which dehydrates the cornea, and consequently contributes to thinning (Mazzotta & Caragiuli, 2014; Rechichi et al., 2016). An evaporative effect resulting from the de‐epithelialised cornea also contributes to further thinning (Iwata et al., 1969). In fact, avoiding the use of an eyelid speculum during the initial riboflavin soaking period, the technique used in this study, led to a lesser reduction in the corneal thickness (Soeters et al., 2014). In spite of this, 74% of the patients randomised to isoosmolar riboflavin required the addition of SW to maintain a minimum corneal thickness of 400 μm (compared to 15% in the hypoosmolar cohort). Patients in the isoosmolar group were at most risk of needing addition of SW during the irradiation phase, which can be explained by the progressive thinning throughout the CXL procedure in combination with the use of an eye‐lid speculum during the irradiation phase which increases the evaporation from the cornea and further contributes to thinning (Soeters et al., 2014). Therefore, we recommend that corneal pachymetry is used when performing CXL with isoosmolar riboflavin, especially during the irradiation phase. Corneal pachymetry can also be of importance when using hypoosmolar riboflavin if the corneal thickness is close to 400 μm at the beginning of CXL. However, the use of SW cannot be standardised as there is a significant inter‐subject difference in the thinning during the CXL procedure and the addition of SW must thus be individualised.

Difficulties in maintaining an adequate corneal thickness could explain why isoosmolar riboflavin is rarely used in clinical practice. In fact, according to the survey on current clinical practice in four Nordic countries (Gustafsson, Vicente, et al., 2023), only one centre of 19 (5%) used dextran‐containing isoosmolar riboflavin only. This is similar to the findings of a survey conducted in the United Kingdom, in which only one centre of 16 (6%) used this kind of riboflavin (Hayes et al., 2023). The reason for the limited use of isoosmolar riboflavin is unknown but could be related to difficulties in maintaining an adequate corneal thickness during the CXL procedure. In contrast to clinical practice, isoosmolar riboflavin is commonly used as a reference in scientific investigations (Ng et al., 2021). However, its use in scientific studies is questionable due to difficulties in using isoosmolar riboflavin in a standardised way. In the Nordic survey, 9 of 19 centres (47%) used hypoosmolar riboflavin as the only kind of riboflavin or as an additive to the dextran‐containing isoosmolar riboflavin. Although it is frequently used in clinical practice, there is a lack of RCTs evaluating the efficacy and safety of hypoosmolar riboflavin.

It is not known whether the addition of SW affects the outcome in terms of efficacy and safety. This treatment strategy is, however, common according to the survey of the Nordic countries, as 32% of the centres reported the use of SW in cases of thin corneae (Gustafsson, Vicente, et al., 2023). On the one hand, diluting the riboflavin in the stroma could lead to a lower CXL effect and less shielding, especially when hypoosmolar riboflavin is used (Wollensak & Spörl, 2019), with negative effects on the endothelial cells. On the other hand, as the swelling of the cornea occurs mainly in the sparse posterior stroma and not in the densely packed anterior stroma where most of the CXL effect is found (Kohlhaas et al., 2006; Wollensak, 2010; Wollensak & Spörl, 2019), similar biomechanical outcomes could be expected. Also, increasing corneal thickness reduces the amount of irradiation at the endothelial level. So far, it is unclear whether the necessary interruption of the irradiation process during the addition of SW and its diluting effect on the riboflavin may decrease the crosslinking effect. Only one study has been published using hypoosmolar riboflavin with and without the addition of SW (Gustafsson et al., 2017), suggesting that there were no differences in the treatment efficacy 1 year after CXL, measured in terms of the reduction of Kmax. However, no assessment of the endothelial cell density was performed.

The use of SW is an interesting alternative to other CXL protocols used in cases of thin corneae (Padmanabhan & Dave, 2013), such as the contact‐lens‐assisted CXL protocol (Jacob et al., 2014). In this protocol, the corneal thickness is artificially increased by a contact lens. However, the reduction in oxygen availability due to the presence of the contact lens must be considered (Richoz et al., 2013) and the biomechanical effect is reported to be one third less than with the standard CXL protocol (Wollensak et al., 2019). The use of SW to increase the corneal thickness could also be an alternative to the “sub‐400 protocol” (Hafezi et al., 2021) in which the UVA irradiation is reduced according to the thickness of the cornea. A limitation of the SW protocol is that it cannot be standardised to all patients and future investigations evaluating its efficacy should stratify according to how many minutes SW is administered. Also, overall it prolongs the treatment time due to the interruption of the irradiation. However, similar to the sub‐400 protocol, a strength of the SW protocol is its ability to be tailored to the specific needs of each individual patient.

One treatment‐associated side effects were encountered during this study as one patient developed clinical signs of bacterial keratitis. The use of a soft contact lens after CXL is also associated with a higher risk of microbial keratitis (Tzamalis et al., 2019). Thus, we decided to interrupt the use of a soft contact lens during this study. Intuitively, a contact lens could be expected to promote epithelial healing as it protects the cornea from mechanical trauma resulting from the eyelids and to reduce pain. However, we saw no side effects in terms of delayed epithelial healing or more pronounced post‐operative pain. These findings are in line with those from a previous study where no significant differences were found in epithelial healing or in pain score in patients with and without a soft contact lens after CXL (Soeters et al., 2020).

A strength of this study is the prospective randomised design and the inclusion of corneae thinner than 400 μm. A limitation is that an increase of Kmax of 0.5 D over 6 months or 1.0 D over 12 months was used as detection limits to diagnose progressive keratoconus. This is due to a lack of knowledge of its limitations at the time the study was designed. More recent publications have demonstrated the importance of using detection limits based on inter‐day repeatability data and considering the severity of keratoconus in the measured patients (Gustafsson et al., 2020; Gustafsson, Bergström, et al., 2021; Gustafsson, Faxen, et al., 2021). Another limitation is that a handheld pachymeter was used to assess intraoperative corneal thickness. An intraoperative OCT device would have been a better alternative (Hassan et al., 2014); however, this technology was not available to the authors at the time the study was initiated. Such a technique would have provided a more extensive view of the corneal thinning including the entire corneal profile. It would also have avoided touching the cornea with the pachymeter. Also, the stromal oedema could have affected the measurements by ultrasonography (De Bernardo et al., 2019; Hassan et al., 2014; Kaya et al., 2012). It is noteworthy that there was a male preponderance among the recruited patients. However, this reflects the gender distribution among patients with keratoconus seeking care at our hospital (Gustafsson et al., 2020).

In summary, if the 400 μm safety limit is used, the corneal thickness should be measured during CXL, especially if isoosmolar riboflavin is used. Also, the use of isoosmolar riboflavin in scientific investigations may be suboptimal as it can require intraoperative modulation of the corneal thickness limiting its use in a standardised way. This study also demonstrates that the addition of SW can effectively counteract the thinning effect of riboflavin solutions during the UVA irradiation allowing intraoperative customised modulation of the corneal thickness. The effect of this technique on the outcome and safety of CXL will be analysed when the one‐year follow‐up is complete.

## FUNDING INFORMATION

Lions Forskningsfond Skåne, Sweden The Foundation for the Visually Impaired in Former Malmöhus County, Sweden Synoptikfonden, Denmark.
